# A Hospital for Belgian Soldiers

**Published:** 1915-05-15

**Authors:** 


### A Hospital for Belgian Soldiers.

BY AN ENGLISHWOMAN.

I have seen many accounts in your valuable paper of different hospitals at the Front, and think the working of the hospital for Belgian soldiers, only eight miles from the firing line, may prove interesting to your readers.

The hospital, formerly an hotel, lends itself splendidly to its present use. On the ground floor are two operating theatres, radiograph room, laboratory, and a room for cutting up dressings. Further to the front of the building is a large dining room, with kitchens at the side, and cellars downstairs used for stores, etc. On the first floor is a large salon for convalescent patients and staff, overlooking the sea, and made very comfortable with chairs, lounges, a gramophone, and a piano, for the use of the patients.

Above are four floors, with rooms leading off either side of a corridor. On one side small rooms containing one bed only, for very serious cases or officers. On the other, larger rooms, each containing six beds. On each floor there is a service room with cupboards for linen, stores, etc., where the food sent up from the kitchens is served and the washing-up done; a small room used as a report room, a bathroom, lavatory, sluice, and dirty-linen cupboard. This part contains 180 beds.

Surrounding the hospital are villas for sleeping accommodation for the staff, linen room, and laundry, dispensary, and one for fumigation.

The patients are well cared for, have good food, plenty of clean linen, and many of the comforts of a home hospital. On admission their clothes are put into a sack marked with their name, number, and list of contents. These are sent to the villa to be fumigated, brushed, mended, and stored until the patient leaves. Then clean shirt, socks, vest, pants, and, when necessary, a grey flannel suit is added.

The ambulance is under the direction of Dr. Depage, with Madame Morel as directrice, and an Englishwoman, Miss Florence Winch, as matron. The housekeeping is well carried out by a Belgian lady, Madame de Brockdorf. The food is good and well served. The laundry is excellently worked by Madame Jansen, the wife of a doctor, and the fumigation department by another Belgian lady, Madame Lippens. The medical staff consists of eleven Belgian, one English, and one Canadian doctor; the nursing staff of Belgian, Danish, and English sisters; the latter are in the majority, all fully trained, and ably helped by assistants with some training in first aid.

(*Continued on page vi.*)

